# Monosomy 3 Influences Epithelial-Mesenchymal Transition Gene Expression in Uveal Melanoma Patients; Consequences for Liquid Biopsy

**DOI:** 10.3390/ijms21249651

**Published:** 2020-12-17

**Authors:** Andrea Soltysova, Tatiana Sedlackova, Dana Dvorska, Karin Jasek, Pooneh Chokhachi Baradaran, Viera Horvathova Kajabova, Lucia Demkova, Verona Buocikova, Terezia Kurucova, Darina Lyskova, Alena Furdova, Gabriel Minarik, Pavel Babal, Zuzana Dankova, Bozena Smolkova

**Affiliations:** 1 Department of Molecular Biology, Faculty of Natural Sciences, Comenius University in Bratislava, Ilkovicova 6, 841 04 Bratislava, Slovakia; andrea.soltysova@uniba.sk (A.S.); terka.kurucova@gmail.com (T.K.); 2 Institute for Clinical and Translational Research, Biomedical Research Center, Slovak Academy of Sciences, Dubravska Cesta 9, 845 05 Bratislava, Slovakia; 3 Comenius University Science Park, Ilkovicova 8, 841 04 Bratislava, Slovakia; tatiana.sedlackova@gmail.com; 4 Geneton Ltd., Ilkovicova 8, 841 04 Bratislava, Slovakia; 5 Laboratory of Genomics and Prenatal Diagnosis, Biomedical Centre Martin, Jessenius Faculty of Medicine in Martin, Comenius University in Bratislava, Mala Hora 10701/4A, 03 601 Martin, Slovakia; dana.dvorska@uniba.sk (D.D.); karin.jasek@uniba.sk (K.J.); zuzana.dankova@uniba.sk (Z.D.); 6 Department of Molecular Oncology, Cancer Research Institute, Biomedical Research Center, Slovak Academy of Sciences, Dubravska Cesta 9, 845 05 Bratislava, Slovakia; pooneh.baradaran@savba.sk (P.C.B.); viera.kajabova@savba.sk (V.H.K.); lucia.demkova@savba.sk (L.D.); verona.buocikova@savba.sk (V.B.); 7 Department of Genetics, Faculty of Natural Sciences, Comenius University in Bratislava, Ilkovicova 6, 841 04 Bratislava, Slovakia; 8 Department of Ophthalmology, Faculty of Medicine, Comenius University in Bratislava, Ruzinovska 6, 821 01 Bratislava, Slovakia; darina.lyskova@gmail.com (D.L.); alikafurdova@gmail.com (A.F.); 9 Institute of Molecular Biomedicine, Faculty of Medicine, Comenius University in Bratislava, Sasinkova 4, 811 08, Bratislava, Slovakia; gabriel.minarik@gmail.com; 10 Department of Pathology, Faculty of Medicine, Comenius University in Bratislava, Sasinkova 4, 811 08 Bratislava, Slovakia; pavel.babal@fmed.uniba.sk

**Keywords:** uveal melanoma, epithelial-mesenchymal transition, circulating tumor cells, circulating tumor DNA, gene expression profiling

## Abstract

Despite outstanding advances in diagnosis and the treatment of primary uveal melanoma (UM), nearly 50% of UM patients develop metastases via hematogenous dissemination, driven by the epithelial-mesenchymal transition (EMT). Despite the failure in UM to date, a liquid biopsy may offer a feasible non-invasive approach for monitoring metastatic disease progression and addressing protracted dormancy. To detect circulating tumor cells (CTCs) in UM patients, we evaluated the mRNA expression of EMT-associated transcription factors in CD45-depleted blood fraction, using qRT-PCR. ddPCR was employed to assess UM-specific *GNA11*, *GNAQ*, *PLCβ4*, and *CYSLTR2* mutations in plasma DNA. Moreover, microarray analysis was performed on total RNA isolated from tumor tissues to estimate the prognostic value of EMT-associated gene expression. In total, 42 primary UM and 11 metastatic patients were enrolled. All CD45-depleted samples were negative for CTC when compared to the peripheral blood fraction of 60 healthy controls. Tumor-specific mutations were detected in the plasma of 21.4% patients, merely, in 9.4% of primary UM, while 54.5% in metastatic patients. Unsupervised hierarchical clustering of differentially expressed EMT genes showed significant differences between monosomy 3 and disomy 3 tumors. Newly identified genes can serve as non-invasive prognostic biomarkers that can support therapeutic decisions.

## 1. Introduction

Uveal melanoma (UM), comprising approximately 83% of ocular and 3% of all melanomas, is the most frequent intraocular tumor in adults [1]. The average annual incidence alters broadly according to ethnicity, age, or latitude, with the highest numbers in white Caucasians [2]. The UM tumors derive from the uveal layer of the eye; the choroid is the most frequent location (82%), with remaining cases originating from the ciliary body (15%) and iris (3%) [3]. Primary UM is treated with either surgery or radiation with a low local recurrence rate. Most UM patients survive less than 12 months after metastases diagnosis by virtue of no efficient therapies for metastatic UM [4]. Various clinical, pathological, molecular, and cytogenetic markers assessed in tumors can predict the risk of metastases and survival. Clinical and histopathological factors associated with poor prognosis include extra-vascular matrix pattern, epithelioid melanoma cytomorphology, high mitotic rate, and inflammatory infiltration, although their sensitivity and specificity are limited [5].

Cytogenetic aberrations, the most serious being the loss of one copy of chromosome 3 (M3) along with 6p gains, 8p loss/8q gain, and 1p deletion, also carry prognostic significance [6]. The loss of function mutations in the BRCA1 associated protein (*BAP1*) gene, located on 3p21, were predominantly determined in M3 tumors, implying that *BAP1* abnormalities are significantly correlated with the development of metastases in UM patients [7]. Gene expression profiling can predict metastatic risk with a higher probability than clinical stage or chromosome 3 status. Expression panel of 15 genes, by Castle Biosciences, which categorizes UM patients as Class 1 (low metastatic risk) or Class 2 (high metastatic risk) are commercially accessible now [8]. However, besides not very specific clinicopathological characteristics, no prognostic biomarkers are available for patients who are treated by stereotactic radiosurgery.

UM tumors tend to metastasize via the hematogenous route to remote body areas, notably to the liver. There is a dormancy between the primary tumor treatment and the initiation of metastases differing from months to decades [9]. Transcription factors regulating the epithelial-mesenchymal transition (EMT) program contribute to carcinogenesis and the formation of metastasis in various tumors, including UM. EMT is the process in which primary tumor cells gain migratory and invasive characteristics and become circulating tumor cells (CTCs) by losing their cell-cell adhesion and cell polarity. The EMT program is regulated by various zinc-finger transcription factors, including the Snail family transcription repressors 1 and 2 (*SNAI1* and *SNAI2*) [10], zinc finger E-box-binding protein 1 and 2 (*ZEB1/2*) [11,12], or by helix–loop–helix transcriptional regulators, like twist family bHLH transcription factor 1 (*TWIST1*) [13]. It has been experimentally proven that the down-regulation of *ZEB1*, *TWIST1*, and *SNAI1* in vitro decreases the invasive properties of UM cells [14].

UM patients with the surgically treated primary disease can develop metastases later, indicating that UM cells have already disseminated into the circulation earlier than the primary tumor was diagnosed. Taking this into account, one would anticipate that CTCs should be easily detectable at the time of diagnosis. However, although CTCs are primarily distinguished in the blood of metastatic UM patients, in the majority of patients with primary UM, CTCs are undetectable by currently available methods [15]. Meanwhile, it is unknown whether the reason is the seeding of CTCs by metastatic lesions or the low number of CTCs in the bloodstream at the time of diagnosis. Notwithstanding, the clinical value of CTCs or circulating tumor DNA (ctDNA) has been recently explored in several cancers as a prognostic biomarker [16]. Various techniques are available to assess ctDNA; among them, the most specific method is the detection of the specific gene mutations harbored by the tumor. About 95% of UM-specific mutations occur in G protein subunit alfa q (*GNAQ*), G protein subunit alfa 11 (*GNA11*), cysteinyl leukotriene receptor 2 (*CYSTLR2*), and phospholipase C beta 4 (*PLCB4*) genes [17,18].

As very little is known about EMT’s molecular nature in mesenchymal tumors, including UM, the whole-genome gene expression approach was applied with a focus on EMT-associated genes to clarify the role of EMT in hematogenous dissemination and to identify new, prognostically relevant, differentially expressed genes. Moreover, we assessed the presence of CTCs and ctDNA in the peripheral blood of primary and metastatic UM patients, focusing on the detection of traditional EMT-associated transcription factors (TFs) and driver mutations, previously associated with UM development and progression. These results can help to a better understanding of the factors contributing to hematogenous dissemination in UM.

## 2. Results

### 2.1. Clinico-Pathological Characteristics of Patients

In the present study, 53 UM patients were enrolled between August 2018 and September 2020; among them, 10 diagnosed in stage IV. One of the primary UM patients developed metastases 8 months after treatment of the primary tumor (Table 1).

**Figure 1:**
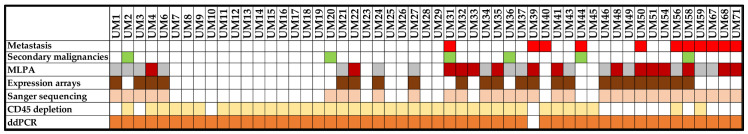
Schematic presentation of the major conducted analyses. Blood was collected for all patients enrolled except for one (n = 52). Tumor tissues were available for 31 patients who underwent enucleation. M3 and D3 status were assessed by MLPA; M3 is highlighted by brown, D3 by grey color. The quality of tumor material in the samples collected after radiotherapy in the past was poor, hampering subsequent MLPA analysis. Sanger sequencing was done in tumor tissues focusing on *GNA11* p.Q209L, p.Q209P, and p.R183C; *GNAQ* p.Q209P, p.Q209L, p.Q209R, and R183Q; *PLCβ4* D630Y, and *CYSLTR2* L129Q mutations. Identical mutations were assessed by digital droplet PCR (ddPCR) in tumor tissues and subsequently in DNA extracted from plasma samples.

Patient sex ratio was similar, with 47.2% (n = 25) being males and 52.8% being (n = 28) females. The median age at the time of diagnosis was 67 years (range 33–87 years). The right eye was affected in 49.1% (n = 26), the left eye in 50.9% (n = 27) of the patients. The median tumor volume was 1.1 cm^3^ (ranging between 0.2–2.6 cm^3^); 71.1% (n = 38) of patients had a tumor volume less than 1.55 cm^3^, while in 28.3% (n = 15) the tumor volume exceeded 1.55 cm^3^. The majority of the tumors, 48.7% (n = 19), were spindle-cell, while 25.6% (n = 10) were classified as epithelioid and the same number as mixed. Altogether 60.4% (n = 32) of patients underwent enucleation without prior treatment; 15.1% (n = 8) underwent enucleation after radiation therapy in the past and 24.5% (n = 13) were treated by stereotactic radiosurgery. Locally advanced disease characterized by vascular cell invasion was diagnosed in 12.8% (n = 5) cases, lymphogenic invasion in 25.6% (n = 10), and perineural spread was detected in 23.1% (n = 9) of patients. Secondary malignancy occurred in six cases, namely auricular tumor, gynecologic cancer, lung carcinoma, prostate cancer, cancer of unknown primary (suspect lipoma confirmed by histopathology as metastasis), and colon cancer in patients UM2, UM20, UM31, UM36, UM44, and UM58, respectively. The presence of secondary malignancies and metastases in individual patient samples are shown in Figure 1.

### 2.2. Multiplex Ligation-Dependent Probe Amplification

**Figure 2:**
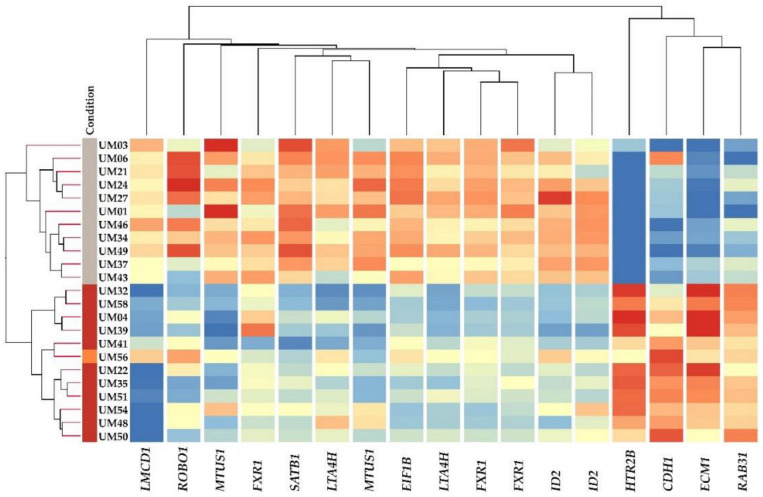
Unsupervised clustering of 12 Class 1 vs. Class 2 discriminating genes. Multiple gene probes were present twice on the chip. Chromosome 3 status is shown on the left side, M3 tumors are highlighted by brown, D3 tumors are highlighted by gray. The UM56 tumor with 1p loss and 8q gain is highlighted by orange. The red color represents up-regulated gene expression, the blue color represents down-regulated gene expression while the yellow represents no change.

MLPA was used to evaluate deletion/duplication status of specific loci located on chromosomes 1, 3, 6, and 8, considered recurrent genetic alterations in UM. As chromosome 3 monosomy strongly correlates with metastatic death, while chromosome 8 gains occur later in UM tumorigenesis, we focus herein on monosomy 3 (M3) only. M3 was detected in 51.6% (n = 16), disomy 3 (D3) in 48.4% (n = 15) of analyzed tumor tissues. Class 2 expression profile was simultaneously detected in all M3 tumors in which gene expression profiling was performed (Figure 2). The only exception was UM56, where the Class 2 gene expression profile was associated with 1p loss and 8q gain, without M3 presence, therefore classified as D3 based on MLPA results.

### 2.3. EMT-Associated TF Expression in Peripheral Blood of UM Patients

**Figure 3:**
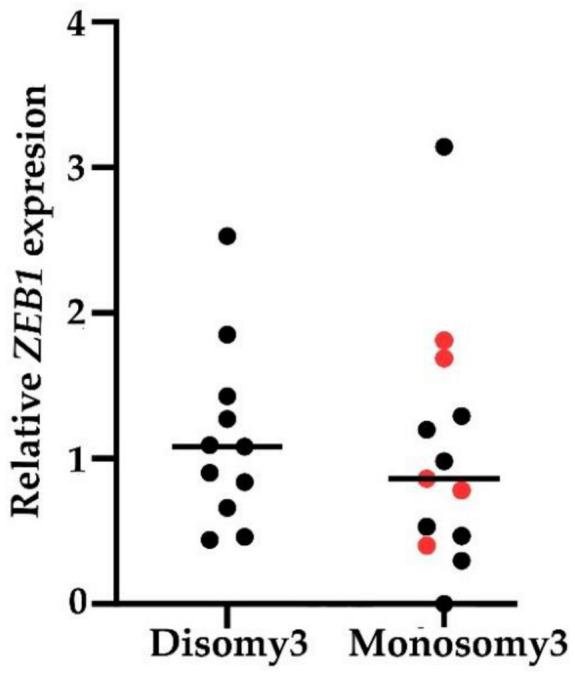
Relative *ZEB1* expression in a CD45-depleted peripheral blood fraction of M3 and five metastatic UM patients (highlighted by red) compared to D3. The difference between D3 and M3 *ZEB1* expression was not significant.

We assessed the gene expression of four EMT-associated TFs, namely *SNAI1*, *SNAI2*, *TWIST1* and *ZEB1*, and epithelial marker keratin 19 *(KRT19)* in a CD45-depleted fraction of 34 primary and five metastatic UM patients’ peripheral blood. CTC enrichment was performed using RosetteSep™ Human CD45 Depletion Cocktail. TaqMan gene expression assays (described in more detail in the Materials and methods paragraph) were used for gene expression analysis. *KRT19* and all studied EMT TFs gene transcripts, except for *ZEB1*, were mostly undetectable in peripheral blood of UM patients (39/39 for *KRT19* and *SNAI2*, 33/39 for *SNAI1,* and 22/39 for *TWIST1*). Although gene expression of *ZEB1* was higher than the other studied genes, all samples were evaluated as negative compared to cut-off values, set based on the expression of *ZEB1* in CD45-depleted peripheral blood fraction of 60 healthy controls [19]. We also compared the *ZEB1* gene expression of M3 and metastatic patients to those of D3; however, no significant differences were found (Figure 3).

### 2.4. Circulating Tumor DNA

Given the negative results of CTCs detection using CD45-depleted peripheral blood, droplet digital polymerase chain reaction (ddPCR) was used to assess if the analytical sensitivity of RT-PCR or other factors were responsible for negative findings. ddPCR has been proven as a highly sensitive detection method allowing for the identification of rare circulating tumor-specific DNA fragments (ctDNA) in peripheral blood. Eight ddPCR assays were used (their numbers are listed in the material and methods paragraph) to detect nine mutations in four genes, namely, *GNA11* p.Q209L, p.Q209P, and p.R183C; *GNAQ* p.Q209L, p.Q209P, p.Q209R, and p.R183Q; *PLCβ4* p.D630Y, and *CYSLTR2* p.L129Q. These mutations were selected as they have been reported to be present in more than 90% of UM tumors. Firstly, all mutations were interrogated in tumor tissues (available for 31 patients) by ddPCR and then identical mutations were detected in the same tissues using Sanger sequencing to validate assays performance. When 100% agreement was confirmed between the two methods, ddPCR was used to detect the same mutations in peripheral blood of 52 UM patients. *GNA11* p.Q209L and *GNAQ* p.Q209P were the two most frequent mutations. *GNA11* p.Q209L was present in 54.8% (n = 17), while *GNAQ* p.Q209P in 32.3% (n = 10) tumors. *GNA11* p.Q209P, *GNA11* p.R183C, *GNAQ* p.Q209L, and *GNAQ* p.Q209R mutations were identified each in one tumor sample (Table 2).

**Figure 4:**
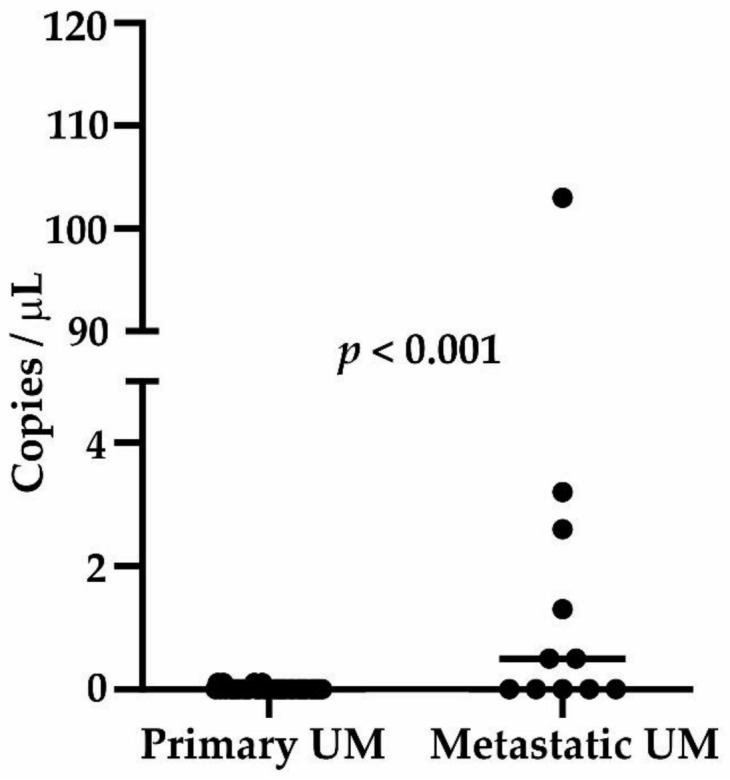
The number of ctDNA copies in µL of plasma in primary UM and metastatic UM patients. The patient UM56 with the highest number of copies had extremely locally advanced disease at the time of diagnosis. The difference in the number of copies between primary and metastatic UM was significant. The *p*-value also remained the same after the outlier (UM56) was excluded from the analysis.

In plasma samples, tumor-specific mutations were identified in 21.4% (n = 9) of patients, only in 9.4% (n = 3) primary UMs, while in 54.5% (n = 6) of metastatic patients. In primary UM patients, the median value was 0 cfDNA copies per µL (minimum = 0, maximum = 0.13), while in metastatic patients the median was 0.3 copies per µL (minimum = 0, maximum = 108). The number of copies per µL was significantly higher in metastatic patients (*p* < 0.001, Figure 4). The patient presenting with an extremely high number of copies (n = 108/µL, UM56) was diagnosed with a locally advanced primary disease after the onset of metastatic disease. The *p*-value remained equally significant even after excluding this extreme value from the analysis. We did not find a correlation between tumor volume and the number of copies.

### 2.5. Gene Expression Profiling of EMT-Associated Genes

**Figure 5:**
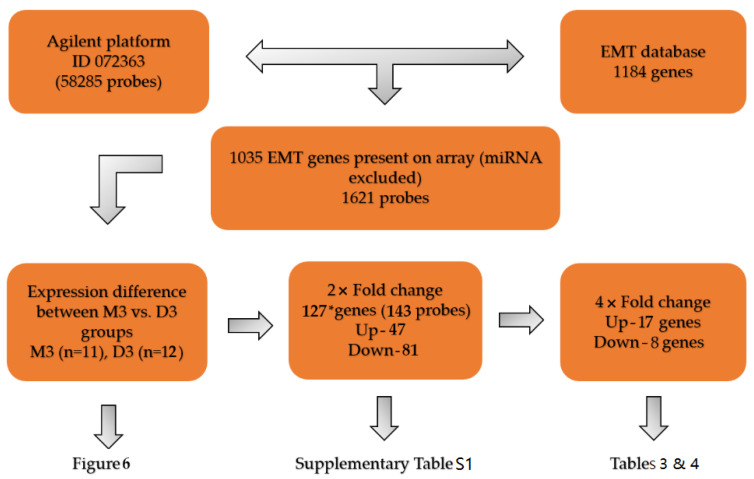
A flow-chart depicting the workflow of the procedure for selecting genes from the EMT database and evaluating the differences in mRNA expression in M3 and D3 tumors. * One gene with opposite regulation of its two transcripts was excluded. Sample UM56 (Class 2 expression profile, monosomy1, D3) was excluded from the statistical analysis.

Finally, the whole-genome mRNA expression analysis was performed in 23 tumor tissues based on chromosome 3 status. The data on EMT-associated genes mRNA expression were acquired from the SurePrint G3 Human Gene Expression 8×60K v2 Microarray (Agilent). Altogether 277 genes (322 probes) among 1184 genes from the EMT gene database (http://dbemt.bioinfo-minzhao.org/) significantly differed, but only 127 genes (143 probes) differed 2-fold or more between M3 and D3 tumors (Figure 5).

**Figure 6:**
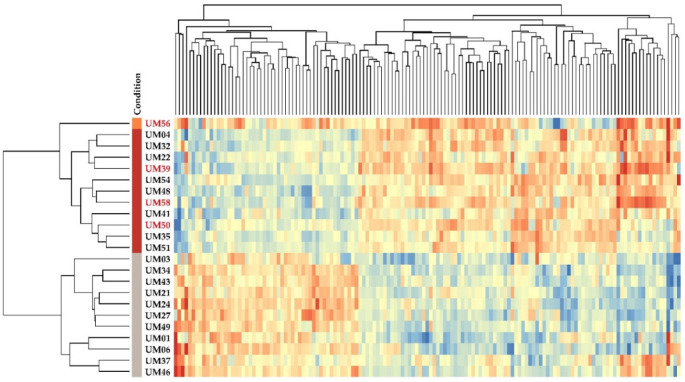
A heatmap of EMT-associated genes mRNA expression in UM tumors with at least 2-fold change. Chromosome 3 status is shown on the left side, M3 tumors are highlighted in brown, D3 tumors are highlighted in gray. UM56 highlighted in orange, a metastatic patient with 1p loss and 8q gain, whose disease status was discussed earlier, showed greater similarity with the M3 group. As the mRNA expression of three other metastatic patients (red numbers) showed a similar pattern as those with primary UM, they were included in the heatmap. The red color represents up-regulated gene expression, the blue color represents down-regulated gene expression, while the yellow represents no change.

Unsupervised hierarchical clustering of differentially expressed EMT genes showed significant differences between M3 and D3 tumors. All M3 and D3 samples clustered together, forming two major distinct clusters (Figure 6), illustrating chromosome 3 loss as the main driving event. A list of all up-regulated and down-regulated genes is provided in Supplementary Table S1. UM56, which is only one sample with 1p loss and 8q gain without M3 in our cohort, showed greater similarity with the M3 group profile.

Among the top 17 differentially expressed up-regulated genes (fold change between 22.7 and 4.1, see Table 3 and Table S1) in M3 vs. D3 were E-cadherin (epithelial) (*CDH1*), v-kit Hardy-Zuckerman 4 feline sarcoma viral oncogene homolog (*KIT*), Twist family bHLH transcription factor 2 (*TWIST2)*, S100 calcium-binding protein A4 (*S100A4*), anaplastic lymphoma receptor tyrosine kinase (*ALK*), protein tyrosine phosphatase type IVA, member 3 (*PTP4A3*) and cyclin D2 (*CCND2*). The most significant up-regulation was found for the chemokine (C-C motif) ligand 18 (pulmonary and activation-regulated) (*CCL18*) gene. In addition to these genes, previously associated with the pathogenesis of UM, several new genes were up-regulated in UM3 tumors.

Between eight top significantly down-regulated genes (fold change between −28.5 and −4.3, see Table 4 and Table S1) are three TFs, namely RAR related orphan receptor gamma (*RORC*), SATB homeobox 1 (*SATB1*), and ETS variant transcription factor 1 (*ETV1*). The majority of down-regulated genes have not been reported in UM yet, and we will discuss their potential function later.

**Figure 7:**
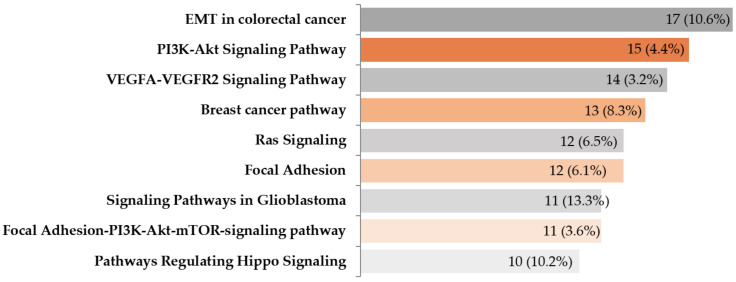
A list of metabolic pathways with 10 and more genes deregulated in M3 tumors selected using GeneSpring software. The numbers in individual columns show the number of genes from our dataset, while the percent represents the proportion of identified genes in a given metabolic pathway.

Multiple metabolic EMT-related pathways were influenced by M3 (Figure 7). The most significant associations (*p* < 10^−5^, see Supplementary Table S2) were identified for 86 pathways. Due to this high number, only pathways where the number of deregulated genes was greater than or equal to 10 (*p* < 10^−9^) are presented in Figure 7. The pathways with the highest *p*-values were signaling pathways in glioblastoma, pathways regulating PI3K-Akt, Hippo, VEGFA-VEGFR2, Ras, and EGFR signaling (*p*-values < 10^−10^). Various cancer-associated pathways such as breast or endometrial, as well as focal adhesion or EMT in colorectal cancer pathways, were also deregulated.

**Figure 8:**
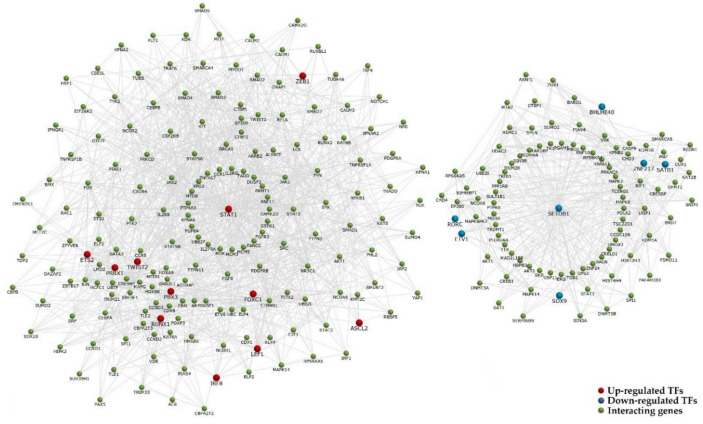
The interaction network of transcription factors differentially expressed in M3. The 11 significantly up-regulated and seven down-regulated TFs in M3 tumors are highlighted by red or blue, respectively.

Interestingly, the mRNA expression of traditional EMT-associated TFs *SNAI1*, *SNAI2,* and *ZEB2* did not differ between M3 and D3 tumors, while *TWIST2* and *ZEB1* were up-regulated in M3. Besides these and *PRRX1* already listed in Table 3, several others were significantly up-regulated, namely *PBX3* (*p* = 9.37 × 10^−6^), *RUNX1* (*p* = 3.79 × 10^−4^), *FOXC1* (*p* = 6.31 × 10^−4^), *IRF8* (*p* = 0.001), *ETS2* (*p* = 0.002), *STAT1* (*p* = 0.002), *LEF1* (*p* = 0.004), *RUNX1* (*p* = 0.003), and *ASCL2* (*p* = 0.03). Additionally to *RORC*, *ETV1*, and *SATB1* listed in Table 4, *SETDB1* (*p* = 9.03 × 10^−5^), *ZNF217* (*p* = 7.39 × 10^−5^), *SOX9* (*p* = 0.001) and *BHLHE40* (*p* = 0.001) were also down-regulated (Table S1). Their interaction network is depicted in Figure 8.

## 3. Discussion

UM, the most frequent primary intraocular tumor in adults, is a rare disease. Although several treatment options are available for primary disease, most extremes are enucleation and stereotactic radiosurgery. Half of the patients develop metastases, in spite of the efficient treatment of the primary disease, and no treatment is accessible to prevent metastatic disease so far [48]. Even though various tumor tissue-based prognostic markers, including gene expression changes and chromosomal rearrangements, have been discovered, they are not available for patients treated with stereotactic surgery or other eye-preserving techniques.

A liquid biopsy is a non-invasive, promising approach in plenty of malignancies, allowing early diagnostics and disease progression monitoring [49,50]. Metastasis in UM arises from hematogenous spread unless tumor cells infiltrate the conjunctival lymphatics [51]. The presence of tumor cells in intra-tumoral blood vessels also found in our study is associated with poor prognosis [52,53]. Using blood-based markers remains challenging in UM due to several impediments. One main reason that made it unfeasible up to now is the extremely low number of CTCs or insufficient concentration of ctDNA in the blood of primary UM patients. It was reported by several authors using different methods that the number of CTC varies from 1 to 5 in 10 mL of venous blood [54,55]. Based on current findings showing a significantly higher amount of CTCs in metastatic patients, we can hypothesize that metastasis seeds CTCs into circulation. Moreover, there is no significant association between the number of detectable CTCs in primary UM and their propensity to metastasize [56,57]. Interestingly, disseminated melanoma cells detected in the bone marrow of 328 UM patients had a positive prognostic value [58]. Therefore, implementing new liquid biopsy methods for UM patients is needed, which would aid therapeutic decisions [51].

Based on this and our previous success with the quantitative PCR (qPCR)-based detection of EMT-associated transcription factors in CD45-depleted peripheral blood fraction of breast cancer patients [19,59,60], we tested the suitability of this approach in UM patients. We compared the gene expression of epithelial marker *KRT19* and four EMT-associated TFs, namely *SNAI1, SNAI2, TWIST1,* and *ZEB1* of the 34 primary and 5 metastatic patients to the expression level of identical genes in the peripheral blood of 60 healthy controls. Except for *ZEB1*, gene expression was mostly undetectable in the majority of patients’ blood, independently of their M stage. *ZEB1* expression of any patient did not exceed the cut-off value. Moreover, it did not differ between M3 and D3 patients. Given our previous findings in breast cancer, these surprising results can be explained by low analytical sensitivity and reduced CTCs seeding in primary UM tumors.

An additional important blood-based marker for monitoring disease progression in UM is ctDNA. A high number of tumor-specific recurrent hot spot mutations allows for tumor-specific ctDNA detection [57]. Therefore, we validated our negative findings in patient plasma samples using the highly sensitive ddPCR method. *GNA11* p.Q209L and *GNAQ* p.Q209P were the most frequent tumor-specific mutations, with four more in the same genes identified each in one patient. Consistent with previous reports, ctDNA was identified more frequently in the metastatic than in the primary disease [61,62]. The presence of ctDNA was reported to be 23% in primary UM and 100% of metastatic patients by Beasley et al., while in our case, it was 9.4% and 54.5%, respectively. This discrepancy can be caused by the different clinicopathological status of enrolled patients in two datasets. Moreover, this approach is hampered by the relatively high number of the mutations to be screened, all requiring plasma DNA. Therefore, the total plasma volume for screening individual mutations remains relatively low, decreasing analytical sensitivity. Our findings show that in contrast to patients with localized UM, the majority of metastatic patients had detectable ctDNA in their plasma, confirming the results reported previously [54,57]. Based on this, the authors concluded that given the low proportion of detectable ctDNA in localized disease, ctDNA could not be an adequate marker for the screening of primary UM patients with a high risk of metastasis. However, it may offer an achievable minimally invasive approach for monitoring metastatic disease progression [57].

By ddPCR, we confirmed the low analytical sensitivity of the qPCR method for the detection of selected epithelial and EMT-associated TFs transcripts in the peripheral blood of UM patients, independently of their M stage status. Therefore, we decided to examine the expression of EMT-associated genes in the tumors of UM patients, given that EMT has been studied rarely in UM and other mesenchymal tumors. While E-cadherin (coded by *CDH1*) is considered a tumor repressor, mesenchymal marker N-cadherin (coded by *CDH2*) is regarded as a tumor facilitator in carcinomas. In the few published reports, traditional EMT-associated TFs, among them *SNAI1/2*, *TWIST1,* and *ZEB1,* were involved in UM pathogenesis. However, unlike in carcinomas, an epithelial-like phenotype, rather than mesenchymal-like (spindle-shaped), has been associated with the risk of poor prognosis. The reverse switch between EMT and mesenchymal-epithelial transition (MET) was negatively correlated with the inhibitor of DNA binding 2 (*ID2*) gene expression in the aggressive UMs [22]. Surprisingly in this regard, overexpression of *TWIST1* and *ZEB1* accompanied by *CDH1* down-regulation and *CDH2* up-regulation were reported in prognostically poor UMs [14]. Therefore, it was hypothesized that EMT-TFs are necessary for UM tumorigenesis, but not for EMT morphology switch [63]. The authors demonstrated in vitro and in vivo, that spindle UM cells were able to convert to epithelioid cells and that higher *ZEB1* expression drives UM progression by inducing cell dedifferentiation, proliferation, invasion, and dissemination without a change in the cell morphology [63]. Moreover, *ZEB1* promoted down-regulation of crucial genes involved in melanocyte differentiation, including *BAP1*, melanocyte inducing transcription factor (*MITF9*), tyrosinase (*TYR*), and tyrosinase-related protein 1 (*TYRP*). Interestingly, *ZEB1* expression was only detected in epithelioid cells showing less pigmentation. *ZEB1* was therefore shown to be the major oncogenic factor in UM progression. To disseminate, UM cells have to undergo a loss of adherence. *CDH1* down-regulation is responsible for cell detachment from each other, while extracellular matrix remodeling is important to allow tumor cell dissemination. It was speculated that epithelioid cells, preferentially expressing epithelial differentiation markers such as cytokeratins, are terminally defined phenotypes with rapid proliferation, quick mobility, high invasiveness, and disseminating abilities. The question remains open if this morphology change is related to the formation of cancer stem cells [63].

Gene expression profiling uncovered an interesting association between poor prognosis, substantiated by the M3/Class 2 expression profile, and deregulated expression of 127 EMT-associated genes. As expected, genes associated with the epithelial-like phenotype (*CDH1*), cell lineage determination and differentiation (*TWIST2*), regulation of cell survival and proliferation, stem cell maintenance (*KIT*), cellular communication, and the normal development and function of the nervous system (*ALK*), cell proliferation, motility, and invasive activity promoting cancer metastasis (*PTP4A3*), and cell cycle regulation (*CCND2*) were up-regulated. Besides these that were previously associated with UM pathogenesis, several new genes were also discovered. Interestingly *PTP4A3* (HR 2.54, 95% CI 2.01−3.20) was associated with the risk of liver metastasis development in UM [64]. Moreover, *PTP4A3* up-regulation increased UM cells’ invasivity and migratory potential [23]. This phenotype was associated with actin microfilament network modifications. Similarly, *S100A4,* a member of S100 family proteins, may function in motility, invasion, and tubulin polymerization regulation. Chromosomal rearrangements and altered expression of this gene have been implicated in tumor metastasis formation [21]. *GDF15* is a divergent member of the TGF-ß superfamily ligands. In normal conditions, it regulates cell survival, proliferation, differentiation, migration, and apoptosis. It can be up-regulated by inflammatory stimuli. Its expression in the plasma of UM patients was associated with metastatic risk [29].

The highest up-regulation was found for the *CCL18* gene, highly expressed also in cutaneous melanoma, breast, ovarian, and other cancers [65]. CCL18 is a T-lymphocyte-attractant, having chemotactic activity for naive T-cells, CD4+, and CD8+ T-cells, and thus may play a role in both humoral and cell-mediated immunity responses [66]. In contrast to other malignancies, the presence of tumor-infiltrating lymphocytes in UM has been associated with poor prognosis [39]. *LGALS3* is another gene involved in immune-suppressive pathways up-regulated following *BAP1* loss [31]. As shown previously, *CSPG4* expression was detected in various human cancers, including the majority of human UMs. Given its low expression in non-cancerous tissues, it was studied as a target for antibody therapies [67,68,69]. Hypoxia induces the expression of *JAG1*, a Notch pathway member, whose pharmacologic inhibition largely blocked the hypoxic induction of invasion in UM. The down-regulation of Jag-1 expression was facilitated by *GNAQ* knockdown [37,38].

Several newly discovered genes have not been previously implicated in UM pathogenesis, including *HTN1, MRC2, VWCE, GJB2, LGALS3,* and *PRRX1*. For example, histatin-1, a product of the *HTN1* gene, counteracted the effects of EMT inducers on the outgrowth of oral cancer cell spheroids, suggesting that it affects processes that are implicated in cancer progression [25]. Endo180 protein, coded by the *MRC2* gene, plays a role in extracellular matrix remodeling and plasticity in tumor cell movement, cooperating with the matrix metalloproteinases. It has been suggested that stabilization of the Endo180-CD147 EMT suppressor complex and targeting of the non-complexed form of Endo180 in invasive cells could have therapeutic benefit in the prevention of cancer progression and metastasis [70]. Up-regulation of the *VWCE* gene (synonym *URG11*) was demonstrated to promote proliferation, migration, and invasion in the prostate, non-small cell lung, and hepatocellular carcinomas [71,72,73]. Moreover, it predicted the poor prognosis of pancreatic cancer by enhancing EMT-driven invasion [34]. Connexins control migration in neural crest and cancer cells, interact with the cytoskeleton, and regulate cell polarity and directional movement. *GJB2* gene product connexin 26 was suggested to promote cancer development by facilitating cell migration and invasion [74]. Among the most interesting findings is the up-regulation of *PRRX1* TF in the UM tissues. Prrx1 has been previously implicated in developmental processes associated with fibroblast behavior. Recently, it was shown that it is an EMT inducer, conferring migratory and invasive properties. Unlike other EMT-associated TFs, the loss of Prrx1 is required for cancer cells to metastasize in vivo in carcinomas [75].

We found eight significantly more than 4-fold down-regulated genes in M3. *RORC* is a T helper 17 (Th17)-associated TF. Th17 are CD4+ cells that produce interleukin 17 (IL-17) and are potent inducers of tissue inflammation and autoimmunity [76]. The *SPP1* gene is one of the developmental genes down-regulated in Class 2 tumors [77]. It is involved in different aspects of tumor biology, including invasion and metastasis [40]. Angiogenin (*ANG*), a member of the ribonuclease A superfamily, together with the *ID2* gene, were negatively associated with liver metastasis in UM (HR 0.42, 95% CI 0.33–0.55; HR 0.48, 95% CI 0.39–0.6, respectively) [64]. *SATB1*, another Class 2 discriminating gene, is the global chromatin organizer and TF. It emerged as a key factor in integrating higher-order chromatin architecture [78]. The role of several other down-regulated genes such as cellular communication network factor 5 (*WISP2),* hook microtubule tethering protein 1 *(HOOK1)*, Cathepsin Z (*CTSZ)*, or ETS variant transcription factor 1 (*ETV1*) in UM has to be investigated. They were deregulated in the breast, hepatocellular, colorectal, and gastric cancers [42,44,46,47]. The majority of these genes have been involved in cell angiogenesis, growth, proliferation, and migration, while CTSZ is histone methyltransferase involved in epigenetic regulation. Many other EMT-associated genes were significantly up-regulated or down-regulated in M3 tumors. They are all listed in Table S1. Their differential expression could serve for the identification of new blood-born prognostic markers for UM patients. Since for further analysis it is necessary to obtain a sufficient amount of RNA from the CD45-depleted fraction of peripheral blood from both controls and patients, we plan to verify this hypothesis in the following study.

Several metabolic pathways were deregulated in M3 tumors, not surprisingly PI3K-AKT, focal adhesion, or the Hippo tumor suppressor pathways. Angiogenesis, a hallmark of cancer, has been shown to play an essential role in UM development. However, antiangiogenic drugs, similarly to targeted therapies or immunotherapies, have shown disappointing results in clinical trials so far. Therefore, combination therapies and novel experimental approaches such as gene therapy or targeting epigenetic modifications may offer possible clues for more effective patient management options in UM.

## 4. Materials and Methods

### 4.1. Patients and Sample Processing

The 53 UM patients, consecutively examined at the Department of Ophthalmology, Faculty of Medicine, Comenius University in Bratislava (University Hospital Bratislava in Slovakia), were enrolled in the study. Among them, 10 were diagnosed in stage IV, when metastases were already present. One of the patients developed metastases 8 months after primary UM treatment (Table 1, Figure 1). Metastases were located in liver (n = 4), lungs (n = 3), spine (n = 1), skin (n = 2) and pelvis (n = 1). Samples were collected between August 2018 and September 2020. The study was approved on December 12th, 2018, by the Ethics Committee of the Ruzinov Hospital Bratislava, number EK/250/2018, and written informed consent was obtained from all patients. The clinico-histopathological data of enrolled patients are summarized in Table 1. All patients were treated for the primary ocular lesion with radiotherapy or/and surgery (enucleation). Peripheral blood samples were obtained before treatment from all patients. Fresh tumor tissues were collected from 37 patients who underwent enucleation, either without (n = 31) or after stereotactic surgery in the past (n = 6). Immuno-histologic features were assessed in formalin-fixed aliquots of the tissues.

### 4.2. Analysis of EMT-Associated TFs Expression in Peripheral Blood

#### 4.2.1. CTCs Enrichment Using CD45 Depletion

The RosetteSep™ Human CD45 Depletion Cocktail (StemCell Technologies, Vancouver, BC, Canada) was used to enrich CTCs from whole blood (10 mL) by depleting CD45+ cells. The antibody cocktail crosslinks unwanted cells to red blood cells (RBCs), forming rosettes. The unwanted cells were then pelleted with the free RBCs when centrifuged over a density centrifugation medium. The enriched cell layer was harvested, and after washing and centrifugation steps, the pellets of CD45-depleted cells were mixed with TRIzolVR LS Reagent (Invitrogen Corporation, Carlsbad, CA, USA) and stored at −80 °C until RNA extraction.

#### 4.2.2. Quantitative RT-PCR

CTCs were detected via the evaluation of mRNA expression of mesenchymal (*TWIST1, SNAIL1, SNAI2, ZEB1*) and epithelial (*KRT19*) gene transcripts by qRT-PCR as described previously [19]. RNA from CD45-depleted cells was extracted with TRIzolVR LS Reagent (Invitrogen Corporation, Carlsbad, CA, USA). The RNA concentration was determined by absorbance readings at 260 nm. The isolated RNA was used as a template directly for the qRT-PCR, where reverse transcription was the initial step of qPCR using Sensicript and Omniscript reverse transcriptase (Qiagen, Hilden, Germany) combination in ratio 1:1. The following TaqMan assays from Life Technologies (Carlsbad, CA, USA) were used: *TWIST1*: Hs00361186_m1; *SNAI1*: Hs00195591_m1; *SNAI2*: Hs00161904_m1; *ZEB1*: Hs01566408_m1; *GAPDH* Hs99999905_m1; and *KRT19*: Hs00761767_s1. The expression levels of epithelial or mesenchymal genes in patient samples were compared to the expression values of 60 healthy donors, enrolled within the other study [19]. The highest expression levels of the *KRT19* and mesenchymal TF gene transcripts relative to that of Glyceraldehyde-3-phosphate dehydrogenase (*GAPDH*) were 3.4 × 10^−3^ (median 2.8 × 10^−6^, range: 0–3.4 × 10^−3^) for *KRT19*, 7.5 × 10^−4^ (median 0, range: 0–7.5 × 10^−4^) for *TWIST1*, 3.8 × 10^−2^ (median 3.1 × 10^−3^, range: 5.0 × 10^−4^–3.8 × 10^−2^) for *SNAIL1* and 1.7 × 10^−1^ (median 1.4 × 10^−2^, range: 2.2 × 10^−3^–1.7 × 10^−1^) for *ZEB1*, while *SLUG* transcripts were not detected in any of the samples from healthy donors.

### 4.3. Mutation Detection

#### 4.3.1. DNA Extraction and Quality Assessment

Approximately 1−1.5 cm^2^ large pieces of tumor tissue were snap-frozen in liquid nitrogen immediately after enucleation. Tissue samples were stored at ™80 °C. Before analysis, tissue was mechanically disrupted using mortar and pestle. DNA was subsequently extracted using *Gentra Puregene Tissue Kit* (Qiagen, Hilden, Germany) according to the manufacturer’s instructions. DNA concentration and absorbance at 280 and 260 nm were measured by spectrophotometry (NanoDrop System; NanoDrop, Minneapolis, MN, USA).

For the liquid biopsy, 10 mL of peripheral blood was collected into an EDTA tube from each patient on the day of treatment. Blood samples were processed up to 4 h after sampling. Two centrifugation steps were applied (1500× *g* for 10 min and 3000× *g* for 10 min) to remove any residual intact blood cells carried over from the first centrifugation step. Plasma samples were then flash-frozen in liquid nitrogen and stored at −80 °C. QIAamp Circulating Nucleic Acid Kit (Qiagen, Hilden, Germany) was used for circulating DNA extraction from 3 mL of plasma with an elution volume 70 µL following manufacturer instructions. DNA concentration was measured by the Qubit™ 2.0 Fluorometer (Qubit™ dsDNA HS Assay Kit, Thermo Fisher Scientific, Waltham, MA, USA).

#### 4.3.2. Mutation Detection in Tumor Samples by ddPCR

To introduce and optimize the ddPCR method, mutation detection was done firstly on DNA extracted from 31 UM tumor tissues using QX100™ Droplet Digital™ PCR system (Bio-Rad Laboratories, Inc., Hercules, CA, USA). We used 8 assays, namely dHsaMVD2010049 for *GNA11* p.Q209L, dHsaMDS961917975 for *GNA11* p.Q209P, dHsaMDS314447910 for *GNA11* p.R183C, dHsaP2010051 for *GNAQ* p.Q209L, as well as for the *GNAQ* Q209R mutation detection, dHsaMDV2516794 for *GNAQ* p.Q209P, dHsaMDS533896396 for *GNAQ* p.R183Q,, dHsaMDS848188535 for *PLCB4* p.D630Y and dHsaMDS821441396 for *CYSLTR2* p.L129Q mutations. Based on the premise that mutations are mutually exclusive, samples with one identified type of mutation have not been further tested for the remaining mutations of interest. The 20 μL PCR mix contained 10 μL ddPCR™ Supermix for probes (no dUTP, Bio-Rad Laboratories, Inc., Hercules, CA, USA), 1 μL predesigned assay with primers and probes (450 nM primers/250 nM probes; Bio-Rad Laboratories, Inc., Hercules, CA, USA), 2 μL (2U) of Tru1I enzyme, nuclease-free water, and template DNA up to 7 μL. The droplets were generated in QX200 Droplet Generator cartridges (Bio-Rad Laboratories, Inc., Hercules, CA, USA) and transferred to a 96-well plate (Bio-Rad Laboratories, Inc., Hercules, CA, USA), sealed in PX1™ PCR Plate Sealer (Bio-Rad Laboratories, Inc., Hercules, CA, USA). The PCR program performed in C1000 Touch™ Thermal Cycler (Bio-Rad Laboratories, Hercules, CA, Inc., USA) was used according to manufacturer’s recommendations, as follows: initial denaturation at 95 °C for 10 min (ramp rate ~2 °C/s), followed by 40 cycles of amplification: 94 °C for 30 s, 55 °C for 1 min (ramp rate ~2 °C/s) Final deactivation of the enzyme was performed at 98 °C for 10 min (ramp rate ~2 °C/s). For mutation interrogation, the QX200 Droplet Reader (Bio-Rad Laboratories, Inc., Hercules, CA, USA) and the QuantaSoft 1.7. software (Bio-Rad Laboratories, Inc., Hercules, CA, USA) using a two-color detection system FAM and HEX were used. Results are expressed in copies/microliter or events/well, respectively. A mutation-positive control DNA, a negative (wild-type) control, and a no template control were included in each run, also used to set the threshold. Droplets are assigned as positive or negative by thresholding based on their fluorescence amplitude. All positive droplets were evaluated above the threshold. To exclude false positivity, we determined a false positive rate (FPR) on wild-type tissue samples. The tests providing less than 3000 droplets were excluded from the analysis.

#### 4.3.3. Sanger Sequencing

To validate assays’ performance, we sequenced all 31 UM tissue samples (Figure 1) to determine the genotypes of the above-mentioned nine primary driver mutations. DNA sequencing was performed on PCR products obtained by amplification with the proprietary designed primers (Table 5) in optimized conditions.

The 20 µL PCR reaction mixture contained components from FastStart^™^ Taq DNA Polymerase, dNTPack (Roche Diagnostics, Basel, Switzerland): 0.4 µL of dNTPs, 2.0 µL of the buffer with MgCl_2_, 0.1 µL of FastStart Taq DNA Polymerase, and 10 pmol/L of each primer (Generi Biotech, s.r.o., Hradec Kralove, Czech Republic). PCR steps were: initial denaturation at 95 °C for 10 min, followed by 40 cycles of amplification steps at 95 °C for 30 s, 54–64 °C (depending on the primer set) for 45 s, 72 °C for 1 min, and a final extension at 72 °C for 10 min. Amplification products were separated by electrophoresis on 1.75% agarose gel stained with GelRed nucleic acid gel stain (Biotium, Fremont, CA, USA) and visualized on a UV transilluminator. PCR products were purified by NucleoSpin^®^ Gel and PCR Clean-up (Machery-Nagel, Duren, Germany). Sequencing primers were the same as those used in the PCR reaction. Sequence reaction was performed using the Big Dye Terminator v1.1 Cycle Sequencing Kit (Applied Biosystems, Foster City, CA, USA) and run on the ABI Prism 3500 Genetic Analyzer (ThermoFisher Scientific, Waltham, MA, USA). In order to exclude analytical errors, all sequencing analyses were carried out on both strands. Obtained sequences were analyzed and compared with reference sequences by the BLAST program (http://blast.ncbi.nlm.nih.gov/Blast.cgi).

#### 4.3.4. Circulating Tumor DNA Detection

After optimizing the method and validating the assays, we analyzed 52 plasma samples by the ddPCR (Figure 1). Identical reaction conditions, procedures, and evaluations were used for tissue analyses. A maximum volume 7 µL of plasma DNA was added to each run. DNA extracted from the plasma of two healthy controls was used as the wild-type control.

### 4.4. MLPA and Data Analysis

The quality of DNA extracted from radiosurgery treated patients in the past was low; therefore, these samples were not used for MLPA analysis. A total of 100 ng of DNA was used to identify chromosomal rearrangements in tumor tissues by SALSA MLPA Probemix P027 Uveal melanoma (MRC Holland, Amsterdam, Netherlands). Besides probes located on chromosomes 1, 3, 6, and 8 (seven probes for 1p, 19 probes for chromosome 3, six probes chromosome 6, and six probes for chromosome 8), the kit contains 12 control probes. The reaction was performed according to manufacturer instructions. After amplification, MLPA products were separated by capillary electrophoresis (Genetic Analyzer 3130XL, Applied Biosystems, Foster City, CA, USA) and analyzed by Coffalyser software (MRC Holland, Amsterdam, The Netherlands).

### 4.5. Microarrays

#### 4.5.1. RNA Extraction and Quality Control

RNA extraction was performed from approximately 34 mg of fresh frozen tumor tissue using the RNeasy Mini Kit (Qiagen, Venlo, Netherlands) following manufacturer instructions. The quality of isolated RNA was analyzed using the Agilent RNA 6000 Nano Kit (Agilent Technologies, Santa Clara, CA, USA), and only RNA samples, where RIN numbers were above 7.5, were selected for subsequent gene expression analysis.

#### 4.5.2. Microarray Assay

Microarray analysis was performed on total RNA isolated from tumor tissues of 23 samples (Figure 1). Also, 100 ng of total RNA was primed with T7-promoter primer, and cDNAs were synthesized using MoMULV reverse transcriptase. cDNA labeling was done using the Quick Amp Labeling kit (Agilent Technologies, Santa Clara, CA, USA). During the amplification process, Cy3 labeled CTP nucleotides were incorporated into cDNA, generating labeled cRNA. Labeled targets were purified in order to remove nonincorporated nucleotides and reaction components using the GeneJET^TM^ RNA Purification Kit (ThermoScientific, Waltham, MA, USA). Subsequently, samples with specific activity above 8 were fragmented by incubation for 30 min at 60 °C using Gene Expression Hybridization Kit (Agilent Technologies, USA) and proceeded to the hybridization step (17 h, 65 °C, 10 rpm), where 600 ng of the sample were applied onto SurePrint G3 Human Gene Expression 8×60K v2 Microarray Slide (Agilent Technologies, Santa Clara, CA, USA). Parts of labeled cRNA, which have bound nonspecifically or not bind at all, were washed away in two washing steps using Gene Expression Wash Buffer Kit (Agilent Technologies, Santa Clara, CA, USA) according to the manufacturer’s instructions. Finally, the microarray slide was scanned at resolution 2 µm using the SureScan Microarray Scanner (Agilent Technologies, Santa Clara, CA, USA).

#### 4.5.3. Image and Data Analysis

TIFF images were converted and processed using Feature Extraction Software 12.0.3.2 (Agilent Technologies, Santa Clara, CA, USA). Acquired data of spot intensities corresponding to each sample were imported into GeneSpring 14.9 GX software (Agilent Technologies, Santa Clara, CA, USA), where gene expression differences were analyzed. Statistical analysis using moderate *t*-test was performed to detect changes in gene expression between two groups (M3 vs. D6).

The EMT gene list included in our analysis was from the EMT gene database (http://dbemt.bioinfo-minzhao.org/). The array contains all protein-coding genes; only miRNAs were excluded. We considered all different probes for selected genes, e.g., different transcripts (ID 072363). Several genes were selected repeatedly, as they are encoded by different probes in the Agilent platform.

### 4.6. Statistical Analysis

Statistical analyses were performed using the IBM SPSS statistics version 23.0 software for Windows (IBM Corp. Released 2015. IBM SPSS Statistics for Windows, Version 23.0. Armonk, NY: IBM Corp.) The Sapiro-Wilk test was used to assess the normality of data. Depending on the data distribution, the Mann-Whitney U test or *t*-test was used to assess differences between primary UM and metastatic patients. The categorical variables were tested using χ2 or Fisher’s exact test. To calculate tumor volume “TV = π/6 × (largest basal diameter × width × prominence)” we used the following formula by Gass (Gass 1985).

## 5. Conclusions

Hematogenous dissemination is believed to be an early event and the major cause of metastatic spread in UM. CTCs have been detected with different success in primary and metastatic patients. Intriguingly, disseminated CTC did not correlate with the prognosis. Therefore, revealing the factors involved in the hematogenous spread of UM would significantly contribute to the development of new therapeutic approaches aimed at prolonging dormancy and delaying the onset of metastatic disease in high-risk patients.

## Figures and Tables

**Table 1:** Clinico-pathological characteristics of the included patients.

	All n (%)	Metastasis Absent n (%)	Metastasis Present n (%)	*p*-Value
Gender				
Male	25 (47.2)	22 (52.4)	3 (27.3)	0.138
Female	28 (52.8)	20 (47.6)	8 (72.7)	
Eye				
Right	26 (49.1)	21 (50.0)	5 (45.5)	0.788
Left	27 (50.9)	21 (50.0)	6 (54.5)	
Median age (range)	67 years (33–87)	67	65	
Median tumor volume (range)	1.1 cm^3^ (0.2–2.6)	1.0 cm^3^ (0.2–2.6)	1.4 cm^3^ (0.3–2.6)	0.414
<1.55 cm^3^	38 (71.7)	30 (71.4)	8 (72.7)	0.932
≥1.55 cm^3^	15 (28.3)	12 (28.6)	3 (27.3)	
Diagnosis				
C69.3	43 (81.1)	36 (85.7)	7 (63.6)	0.096
C69.4	10 (18.9)	6 (14.3)	4 (36.4)	
Cell type				
Epitheloid	10 (25.6)	5 (16.7)	5 (55.6)	0.060
Spindle	19 (48.7)	16 (53.3)	3 (33.3)	
Mixed	10 (25.6)	9 (30.0)	1 (11.1)	
Melan-A				
Positive	37 (94.9)	28 (93.3)	9 (100)	0.426
Negative	2 (5.1)	2 (6.7)	0 (0)	
S100				
Positive	36 (92.3)	28 (93.3)	8 (88.9)	0.661
Negative	3 (7.7)	2 (6.7)	1 (11.1)	
HMB45				
Positive	36 (92.3)	28 (93.3)	8 (88.9)	0.661
Negative	3 (7.7)	2 (6.7)	1 (11.1)	
Therapy				
Enucleation	32 (60.4) *	22 (52.4)	9 (81.1)	0.031
Enucleation after radiosurgery in the past	8 (15.1)	7 (16.7)	1 (9.1)	
Stereotactic radiosurgery	13 (24.5)	13 (31.0)	0 (0.0)	
Vascular invasion				
Present	5 (12.8)	2 (6.9)	3 (30.0)	0.060
Absent	37 (87.2)	27 (93.1)	7 (70.0)	
Lymphogenic invasion				
Present	10 (25.6)	5 (17.2)	5 (50.0)	0.041
Absent	29 (74.4)	24 (82.2)	5 (50.0)	
Perineural invasion				
Present	9 (23.1)	6 (20.7)	3 (30.0)	0.547
Absent	30 (76.9)	27 (79.0)	7 (70.0)	
Extrabulbar overgrowth				
Present	30 (76.9)	23 (76.7)	5 (55.6)	0.217
Absent	9 (23.1)	7 (23.3)	4 (44.4)	
TNM staging				
I-IIB	30 (56.6)	27 (62.8)	3 (30.0)	0.059
IIIA-IIIC	23 (43.4)	16 (37.2)	7 (70.0)	
MLPA status ^$^				
Monosomy 3	16 (51.6)	10 (43.5)	6 (75.0)	0.124
Disomy 3	15 (48.4)	13 (56.5)	2 (25.0)	

* one metastatic patient was enrolled 31 months after enucleation, tumor tissue was not available; ^$^ only tumor tissues which were not treated by stereotactic radiosurgery in the past were analyzed; Abbreviations: TNM classification, T, size of the tumor; N, involvement on lymph nodes; M, presence of distant metastasis; MLPA, Multiplex Ligation-Dependent Probe Amplification; C69.3, malignant neoplasm of choroid; C69.4, malignant neoplasm of the ciliary body.

**Table 2:** The presence of *GNA11*, *GNAQ*, *CYSLTR2*, and *PLCB4* mutations in tumor tissues and the peripheral blood of primary and metastatic UM patients.

Mutations	All Tumor Tissues n (%)	All Plasma n (%)	Primary UM Plasma n (%)	Metastatic UM Plasma n (%)
Number *	31 (100)	42 (100)	32 (100)	11 (100)
*GNA11* p.Q209L	17 (54.8)	4 (9.5)	1 (3.1)	3 (27.3)
*GNA11* p.Q209P	1 (3.2)	0		
*GNA11* p.R183C	1 (3.2)	1 (2.4)		1 (9.1)
*GNAQ* p.Q209L	1 (3.2)	0		
*GNAQ* p.Q209P	10 (32.3)	4 (9.5)	2 (6.3)	2 (18.2)
*GNAQ* p.Q209R	1 (3.2)	0		
*GNAQ* p.R183Q	0	0		
*CYSLTR2* p.L129Q	0	0		
*PLCB4* p.D630Y	0	0		
Total	31 (100)	9 (21.4)	3 (9.4)	6 (54.5)

* Valid % were used for individual categories.

**Table 3:** A partial list of up-regulated EMT-associated genes in M3 tumors.

Gene (n = 17)	Chromosome Location	*p*-Value	Fold Change	Function	Reference
*CCL18*	17q12	1.44 × 10^−6^	22.7	Expressed in retinal pericyte UM cells, maintain blood-retinal barrier integrity in UM	[20]
*S100A4*	1q21.3	2.19 × 10^−5^	7.9	Role in motility, invasion, tubulin polymerization, its chromosomal rearrangements and expression changes implicated in tumor metastasis	[21]
*CDH1*	16q22.1	1.05 × 10^−5^	7.7	↑ in Class 2 UM tumors, associated with transcriptional up-regulation, and plasma membrane colocalization	[22]
*PTP4A3*	8q24.3	1.45 × 10^−6^	6.4	↑ the aggressiveness of human UM, ↑metastatic risk in UM patients	[23,24]
*HTN1*	4q13.3	0.031	7.1	↑ epithelial and endothelial cell adhesion and barrier function	[25]
*ALK*	2p23.2-p23.1	0.001	6.0	Role in cellular communication and the normal development and function of the nervous system	[26]
*GJB2*	13q12.11	0.006	5.6	Gap junction channel protein having a role in direct communication between cells	[27]
*MRC2*	17q23.2	8.44 × 10^−5^	5.4	Role in extracellular matrix remodeling by mediating the internalization and lysosomal degradation of collagen ligands	[28]
*GDF15*	19p13.11	0.003	5.2	A serum marker for metastases in UM	[29]
*KIT*	4q12	9.62 × 10^−6^	5.1	↑ in UM, role in cell survival, proliferation, melanogenesis, and hematopoiesis	[30]
*TWIST2*	2q37.3	3.31 × 10^−7^	5.1	Role in the progression of non-epithelium-derived tumors, including melanomas	[14]
*LGALS3*	14q22.3	6.61 × 10^−5^	4.9	↑ in BAP1 loss UM tumors and in metastatic liver of UM patients	[31,32]
*PRRX1*	1q24.2	2.77 × 10^−5^	4.7	Regulates differentiation of mesenchymal precursors	[33]
*VWCE*	11q12.2	1.39 × 10^−9^	4.5	Correlated with poor prognosis of pancreatic cancer by ↑EMT-driven invasion	[34]
*CCND2*	12p13.32	0.002	4.4	Drives cell cycle progression, associated with tumorigenesis	[35]
*CSPG4*	15q24.2	8.44 × 10^−8^	4.3	↑ expression in tumor, perivascular, and oligodendrocyte cells, involved in cell adhesion and migration	[36]
*JAG1*	20p12.2	8.10 × 10^−9^	4.1	↑ expression as response to hypoxia in metastatic spread of UM	[37,38]

Genes with fold change equal to or higher than 4 are included. Abbreviations: *VWCE*—von Willebrand factor C and EGF domains, *HTN1*—Histatin 1, *MRC2*—Mannose receptor C type 2, *GDF15*—Growth differentiation factor 15, *CSPG4*—Chondroitin sulfate proteoglycan 4, *GJB2*—Gap junction protein beta 2, *LGALS3*—Galectin—3, *PRRX1*—Paired related homeobox 1, *JAG1*—Jagged canonical notch ligand 1, ↑ indicates increase.

**Table 4:** A partial list of down-regulated EMT-associated genes in M3 tumors.

Gene (n = 8)	Chromosome Location	*p*-Value	Fold Change	Function	Reference
*RORC*	1q21.3	4.23 ×10^−7^	−28.5	Th17-associated transcription factor	[39]
*SPP1*	4q22.1	2.37 × 10^−6^	−15.4	↓ in M3/BAP1-negative tumors, expressed in hepatic metastases from UM, ↑ serum levels correlate with metastatic melanoma to the liver	[40,41]
*WISP2*	20q13.12	1.26 × 10^−4^	−8.1	Regulates diverse cellular functions, including cell adhesion, migration, proliferation, differentiation	[42]
*ANG*	14q11.2	2.04 × 10^−9^	−7.5	Induce tumor angiogenesis, ↑ cell survival, proliferation and/or migration	[43]
*HOOK1*	1p32.1	1.23 × 10^−8^	−7.2	Involved in endocytic membrane trafficking to the microtubule cytoskeleton	[44]
*SATB1*	3p24.3	9.28 × 10^−9^	−5.1	One of the 12 gene expression profile predictive of primary UM metastasis	[45]
*CTSZ*	20q13.32	0.013	−4.6	Specific functions in cancer invasion and metastasis	[46]
*ETV1*	7p21.2	3.56 × 10^−5^	−4.3	Role in cell growth, angiogenesis, migration, proliferation, and differentiation	[47]

Abbreviations: *SPP1*—secreted phosphoprotein 1; *WISP2*—Cellular communication network factor 5, *ANG*—Angiogenin, *HOOK1*—Hook microtubule tethering protein 1, *CTSZ*—Cathepsin *Z,* ↑ indicates increase, ↓ indicates decrease.

**Table 5:** Sequences of the PCR and sequencing primers.

Genes	Strand	Sequence (5′-3′)
*GNA11* p.Q209P/L	Forward	TCTCTGAGAGCGTCCTTGC
	Reverse	GACTTGGTCGTATTCGCTGAG
*GNA11* p.R183C	Forward	TGGTTTGGGTGCTGTGTC
	Reverse	CGGAAGATGATGTTCTCC
*GNAQ* p.Q209P/L/R	Forward	CATTGCCTGTCTAAAGAACAC
	Reverse	TGCAGAATGGTCGATGTAGG
*GNAQ* p.R183Q	Forward	ATTCGATGATCCCTGTGGTG
	Reverse	ACAGCTTTGGTGTGATGGTG
*PLCB4* p.D630Y	Forward	CAGTGAACTGTGATCTTAAGC
	Reverse	ATCTGGCAGCCAGCGTTC
*CYSLTR2* p.L129Q	Forward	ATCTCCTGTTCATAAGCACGC
	Reverse	AACGCACAACACTCAGCACG

## Supplementary Materials

Supplementary materials can be found at https://www.mdpi.com/1422-0067/21/24/9651/s1.

## Abbreviations

ALK	Anaplastic lymphoma receptor tyrosine kinase
ANG	Angiogenin
BAP1	BRCA1 associated protein
CCL18	C-C motif chemokine ligand 18
CCND2	Cyclin D2
CDH1	E-cadherin
CNVs	Copy number variations
CSPG4	Chondroitin sulfate proteoglycan 4
CTCs	Circulating tumor cells
ctDNA	Circulating tumor DNA
CTSZ	Cathepsin Z
CYSLTR2	Cysteinyl leukotriene receptor 2
ddPCR	Droplet digital PCR
D3	Disomy of chromosome 3
EMT	Epithelial-mesenchymal transition
ETV1	ETS variant transcription factor 1
FPR GAPDH	False positive rate Glyceraldehyde-3-phosphate dehydrogenase
GDF15	Growth differentiation factor 15
GJB2	Gap junction protein beta 2
GNAQ	G protein subunit alfa q
GNA11	G protein subunit alfa 11
HOOK1	Hook microtubule tethering protein 1
HTN1	Histatin 1
ID2	Inhibitor of DNA binding 2
IL	Interleukin
JAG1	Jagged Canonical notch ligand 1
LGALS3	Galectin
KIT	V-kit Hardy-Zuckerman 4 feline sarcoma viral oncogene homolog
KRT19	Epithelial marker keratin 19
MET	Mesenchymal-epithelial transition
MITF9	Melanocyte inducing transcription factor
MLPA	Multiplex Ligation-Dependant Probe Amplification
MRC2	Mannose receptor C type 2
M3	Monosomy of chromosome 3
p	Short arm of chromosome
PLCβ4	Phospholipase C beta 4
POSTN	Periostin
PRRX1	Paired relaxed homeobox 1
PTP4A3	Protein tyrosine phosphatase type IVA, member 3
RBCs	Red blood cells
RORC	RAR related orphan receptor gamma
RT-PCR	Real-time PCR
q	Long arm of chromosome
SATB1	SATB homeobox 1
SLUG	Snail family transcriptional repressor 2
SPP1	Secreted phosphoprotein 1
SNAI1	Snail family transcription repressors 1
SNAI2	Snail family transcription repressors 2
S100A4	S100 calcium-binding protein A4
TF	Transcription factor
TYR	Tyrosinase
TYRP	Tyrosinase-related protein 1
TWIST1	Twist family bHLH transcription factor 1
TWIST2	Twist family bHLH transcription factor 2
UM	Uveal melanoma
VWCE	von Willebrand factor C and EGF domains
WISP2	Cellular communication network factor 5
ZEB1	Zinc finger E-box-binding homeobox 1
ZEB2	Zinc finger E-box-binding homeobox 2
